# Sub-species diversity of *Xanthomonas euvesicatoria* Bulgarian and Macedonian strains from pepper

**DOI:** 10.1080/13102818.2014.947722

**Published:** 2014-10-16

**Authors:** Taca Vancheva, Mariya Stoyanova, Martina Tatyozova, Nevena Bogatzevska, Penka Moncheva

**Affiliations:** ^a^ Faculty of Biology, Sofia University ‘St. Kliment Ohridski’, Sofia, Bulgaria; ^b^ Institute of Soil Science, Agrotechnologies and Plant Protection ‘Nikola Poushkarov’, Sofia, Bulgaria

**Keywords:** bacterial spot of pepper, Bulgarian and Macedonian populations, genotypic and phenotypic variations, RAPD analysis, *Xanthomonas* pathogens

## Abstract

Sub-species diversity of pepper populations of *Xanthomonas euvesicatoria* in Bulgaria and Macedonia in 2012 was the object of this study. Species determination of 44 strains was performed by molecular methods using two pairs of species-specific primers and RFLP (restriction fragment length polymorphism) analysis of the 16S-23S ITS region with *Hpa*II. The populations were characterized by genotypic and phenotypic properties. The genotypic diversity of the strains was evaluated by RAPD (random amplified polymorphic DNA) technique. Primer CUGEA-6 differentiated the strains in two groups, one of which included only Bulgarian strains and revealed a mixed profile of the type strain. Biolog^TM^ metabolite profiles separated the strains in four groups: two of which were composed only of Bulgarian or Macedonian strains. Correlation between the RAPD and the metabolic profiles was observed. Twelve antibiotics and copper ions in five concentrations (1–5 g kg^−1^) were tested for biological activity. The inhibition zones of the Bulgarian strains were statistically proven to be considerably larger than the Macedonian ones in the tests with kanamycin, streptomycin, polymyxin B sulphate, tetracycline and vankomycin. The inhibition zones of the Bulgarian strains were statistically proven to be relatively larger than the Macedonian ones in the copper tests. Based on our studies the Macedonian population of *X. euvesicatoria* manifested a relative homogeneity while a greater diversity was observed in the Bulgarian population.

## Introduction

Bacterial spot of pepper caused by the pathogens of genus *Xanthomonas* has become a very important factor affecting pepper production all over the world. The disease is a major problem in production of bioproducts for fresh consumption and processing especially in areas with high humidity. First records of bacterial spot in Bulgaria were described of tomato in 1936.[1] In the period 1989–1999 *Xanthomonas axonopodis* pv. *vesicatoria* and *Xanthomonas vesicatoria* were intensely studied as one of the main pathogens of tomato.[2,3] In recent years, bacterial spot raises as an economically important disease of pepper.[4] Studies in Macedonia also disclosed the wide distribution of *X. campestris* pv. *vesicatoria* in pepper plants in the country with losses reaching 10%–20% per year.[5–7]

For many years, it was believed that the disease was caused by a single, relatively homogenous pathogenic species – *X. campestris* pv. *vesicatoria*.[8] Later, in the 1990s Stall et al. [9] and Vauterin et al. [10] found that the species contains two genetically and phenotypically distinct groups (A and B). Vauterin et al. [10] suggested reclassification of the xanthomonads and separated *X. campestris* pv. *vesicatoria* into two groups: group A – *X. axonopodis* pv. *vesicatoria* and group B – *X. vesicatoria*. Two other groups (C and D) were later characterized.[11] Jones et al. [12] discovered that groups A, C and D had <70% DNA relatedness with each other, with the type strain of *X. axonopodis* and with other species of *Xanthomonas* genus. Therefore, they renamed group A as *X. euvesicatoria*, group C as *X. perforans* and group D as *X. gardneri*. Group B remained as *X. vesicatoria*.

Although the four species were clearly differentiated, the problem with the control of bacterial spot remains unresolved. The classical approach includes treatment with copper pesticides but in the years their effectiveness has become inversely related to the frequency of their use. One reliable and effective method for control of the disease is breeding pepper varieties with genetic resistance.[13] However, various investigations showed that there is a sub-species variation based on the geographical area. One of the key prerequisites for disease management in each geographical area is the accurate diagnostics, identification of the pathogen and determination of the phenotypic and genotypic diversity in the pathogen populations.

The detection and diagnostics of the pathogen generally include cultivation on semi-selective media and serological tests.[14,15] Since the four species – agents of bacterial spot of pepper have been only recently determined – the molecular techniques for identification are still in a process of development. Analyses of the restriction fragment length polymorphism (RFLP) have provided a highly sensitive strategy for detection of some xanthomonads.[16] In the years, amplification reaction using random oligomeric primers (RAPD-PCR) has been intensively used to reveal genetic variations in various *Xanthomonas* species.[17–19] Species-specific primers have only recently been designed on the basis of the sequence data following amplification fragment length polymorphism (AFLP) analysis.[20–22]

The aim of this study was to investigate the phenotypic and the genotypic diversity of the pepper populations of *X. euvesicatoria* in Bulgaria and Macedonia in 2012. The data obtained are necessary for effective breeding, introduction, and use of resistant pepper varieties and lines which are irreplaceable elements in the development of effective disease control strategies in the specific geographical conditions in Bulgaria and Macedonia.

## Materials and methods

*Strains*. Forty-four bacterial strains originating from Bulgaria and Macedonia were the object of this study. The strains were isolated in 2012 from pepper plants with bacterial spot, possessed pathogenic potential upon artificial inoculation and shared the basic characteristics of genus *Xanthomonas* (Gram reaction, oxidase, inability to grow anaerobically, colonies on YDC). The type cultures *X. vesicatoria* NBIMCC 2427 (= DSM-22252), *X. euvesicatoria* NBIMCC 8731 (= DSM-19128), *X. perforans* NBIMCC 8729 (= DSM-18975) and *X. gardneri* NBIMCC 8730 (= DSM-19127) were used.

### Primers and polymerase chain reaction (PCR) conditions

The taxonomical position of the strains was determined by species-specific PCR with two pairs of primers for *X. euvesicatoria* – Xeu 2.4/Xeu 2.5 and Bs-XeF/Bs-XeR (Table 1), and RFLP analysis of 16S-23S ITS region with *Hpa*II. Sub-species diversity was evaluated by a random amplified polymorphic DNA (RAPD) analysis.

**Table 1.** Sequences of oligonucleotide primers used in PCR amplifications.

Primer	Oligonucleotide sequence (5^′^ → 3^′^)	Reference
Xeu 2.4	CTGGGAAACTCATTCGCAGT	[21]
Xeu 2.5	TTGTGGCGCTCTTATTTCCT	[21]
Bs-XeF	CATGAAGAACTCGGCGTATCG	[20]
Bs-XeR	GTCGGACATAGTGGACACATAC	[20]
16S-2	CTTGTACACACCGCCCGTC	[23,25]
23S-7	GGTACTTAGATGTTTCAGTTC	[23,25]
CUGEA-3	GCGGTACCCG	[26]
CUGEA-4	GCGAATTCCG	[26]
CUGEA-5	CGATCGATGC	[26]
CUGEA-6	GGAAGCTTCG	[26]

Bacterial strains were cultivated in Luria-Bertrani Broth at 28 °C, 200 rpm, overnight prior to DNA extraction. Cell density of the bacterial suspension was adapted to OD_600_ = 1. Genomic DNA was extracted by a DNA isolation kit (STS, Ltd.) according to the manufacturer's instructions. Control of yield and purity of the obtained DNA was performed by measuring with a spectrophotometer Nanodrop 2000 (Thermo Scientific) at 230, 260, 280 and 320 nm.

Amplification with primers Xeu 2.4/Xeu 2.5 was carried out in a total volume of 25 μL containing (final concentration) 0.5x Red *Taq* DNA polymerase MasterMix (VWR Int., LLC), 4 pmol of each primer, and 100 ng of template DNA, under the following reaction conditions: a denaturation step at 94 °C for 5 min, followed by 25 cycles at 94 °С for 45 s, 64 °С for 45 s and 72 °С for 45 s, and a final step at 72 °С for 7 min.[20] Amplification with primers Bs-XeF/Bs-XeR was carried out in a total volume of 25 μL containing (final concentration) 0.5x Red *Taq* DNA polymerase MasterMix (VWR Int., LLC), 4 pmol of each primer, and 100 ng of template DNA, under the following reaction conditions: a denaturation step at 94 °C for 5 min, followed by 25 cycles at 94 °С for 30 s, 64 °С for 30 s and 72 °С for 30 s, and a final step at 72 °С for 7 min.[20]

Amplification with primers 16S-p2/23S-p7 was carried out in a total volume of 50 μL, containing 1x buffer, 1.5 mM MgCl_2_, 2 pmol of each primer, 0.15 mM dNTP, 0.4 U Taq polymerase and 100 ng of DNA, under the following reaction conditions: a denaturation step at 94 °C for 300 s, followed by 30 cycles at 94 °С for 45 s, 58 °С for 45 s, and 72 °С for 45 s, and a final step at 72 °С for 7 min.[23]

Amplification with random primers CUGEA-3, CUGEA-4, CUGEA-5 and CUGEA-6 (Table 1) was carried out in a total volume of 25 μL final volume, containing 1x buffer, 2.5 mM MgCl_2_, 50 pmol of each primer, 0.1 mM dNTPs, 0.5 U Taq polymerase and 100 ng of DNA, under the following reaction conditions: a denaturation step at 94 °C for 4 min, followed by 35 cycles at 94 °С for 60 s, 42 °С for 60 s and 72 °С for 90 s, and a final step at 72 °С for 5 min.[24]

### RFLP analysis of PCR products

The model restriction mapping was based on the 16S, 16S-23S ITS and 23S rDNA sequence data in GenBank for *X. euvesicatoria*, *X. vesicatoria*, *X. gardneri* and *X. perforans* and the sequence for 16S-23S ITS rDNA for *X. euvesicatoria* NBIMCC 8731 (Kizheva et al., unpublished data) using the online tool restrictionmapper.org.

16S-23S ITS fragments amplified by PCR with the primer pair 16S-p2/23S-p7 were analysed by restriction endonuclease digestion with *Hpa*II (Fermentas) in a total volume of 25 μL containing 10 μL enzyme mix (containing, final concentrations: 10 U enzyme and 1x Buffer Tango™ in nuclease-free water) and 15 μL PCR product for 3h at 37 °С.

### Electrophoresis

The PCR and restriction products were separated electrophoretically in 1.5% agarose gel in Tris-borate-EDTA (TBE) buffer for 30 min at 100V, stained with ethidium bromide (EtBr) and visualized under UV light. GeneRuler 100 bp Plus DNA Ladder (Fermentas) was used. The gels were analysed by GenoSoft Capture and GenoSoft Imaging software (VWR Int., LLC).

### Biochemical characterization

Metabolic fingerprints of the strains were obtained using GN Microplates of the system BIOLOG^TM^ (Biolog Inc., USA). Procedure was held according to manufacturer's instructions. The results were cluster analysed through the SPSS 16.0 hierarchical cluster analysis procedure by the Ward's method. The matrix of similarity between the isolates was calculated using the Squared Euclidean distance.[25–28]

### Resistance to copper and antibiotics

Resistance to copper and antibiotics was studied by the disk-diffusion method. Bacterial strains evaluated for susceptibility were prepared as bacterial suspensions (cells in physiological solution) adjusted to an optical density of 0.5 McFarland standard (corresponding to 1.5 × 10^7^ CFU mL^−1^). Antibiotics from different groups were used as impregnated filter discs (μg mL^−1^, water solutions) as follows: gentamycin (50), polymixin B sulphate (50), streptomycin sulphate (50), lincomycin hydrochloride (10), vancomycin (50), kanamycin (50), bacitracin (50), ampicillin (50), cephazoline (10), tetracycline (30), sulfamethoxazole-trimethoprim (23.75/1.25) and chloramphenicol 20 μg/mL, absolute alcohol solution. Limit values of susceptibility were according to NCCLS. Copper ions (Cu^2+^ in CuSO_4_) were used as 50 μL solution in concentrations (g kg^−1^) 1, 2, 3, 4, 5. The diameters of the inhibition zones (mm) were measured 24 h after inoculation.

## Results and discussion

### Species determination

The taxonomical position of the strains was determined by the use of PCR with two pairs of species-specific primers for *X. euvesicatoria* and RFLP analysis of 16S-23S ITS region with *Hpa*II.

**Figure 1.**
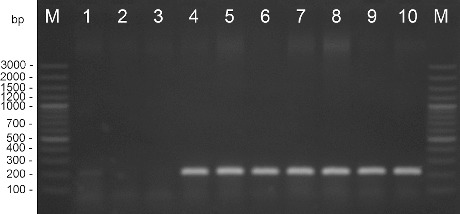
PCR amplification with species-specific primers Xeu 2.4/Xeu 2.5. M – DNA ladder, 1 – *X. perforans* NBIMCC 8729, 2 – *X. gardneri* NBIMCC 8730, 3 – *X. vesicatoria* NBIMCC 2427, 4 – *X. euvesicatoria* NBIMCC 8731, 5–10 – *X. euvesicatoria* strains.

**Figure 2.**
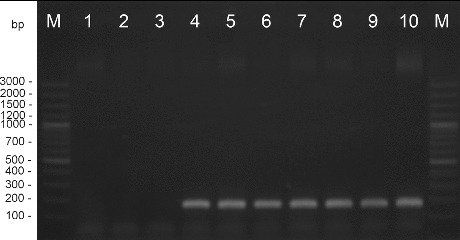
PCR amplification with species-specific primers Bs-XeF/Bs-XeR. M – DNA ladder, 1 – *X. perforans* NBIMCC 8729, 2 – *X. gardneri* NBIMCC 8730, 3 – *X. vesicatoria* NBIMCC 2427, 4 – *X. euvesicatoria* NBIMCC 8731, 5–10 – *X. euvesicatoria* strains.

Amplification with DNA extracted from the pepper strains and type strain *X. euvesicatoria* gave positive results. DNA from the *X. vesicatoria*, *X. gardneri*, and *X. perforans* cultures did not amplify in these reactions. The length of the products was determined as 226 bp for primers Xeu 2.4/Xeu 2.5 and 171 bp for primers Bs-XeF/Bs-XeR (Figures 1 and 2). The obtained results correspond to the results published before.[21,22]

**Figure 3.**
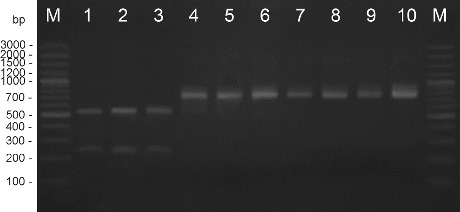
RFLP analysis with endonuclease *Hpa* II. M – DNA ladder, 1 – *X. perforans* NBIMCC 8729, 2 – *X. gardneri* NBIMCC 8730, 3 – *X. vesicatoria* NBIMCC 2427, 4 – *X. euvesicatoria* NBIMCC 8731, 5–10 – *X. euvesicatoria* strains.

Amplification with the primers for 16S-23S ITS region gave a product of 845 bp. The fragment includes ∼150 bp from the 16S rRNA region, ∼550 bp of the ITS 16S-23S region and ∼207 bp from the 23S rRNA region.[23] The RFLP method was evaluated to differentiate between species having closely related identities in the 16S–23S rDNA ITS.[23] Very few data are available on the 16S-23S ITS rDNA for *X. euvesicatoria*. The model restriction mapping of 16S-23S ITS region based on the data in GenBank with endonuclease *Hpa*II did not distinguish the four species. However, the model restriction mapping of 16S-23S ITS region based on the data in GenBank and the sequence of the type strain *X. euvesicatoria* (Kizheva et al., unpublished data) with the same endonuclease distinguished the species *X. euvesicatoria* from *X. vesicatoria*, *X. gardneri*, and *X. perforans* gave one restriction product against two restriction products, respectively (Figure 3).

### Sub-species genotypic diversity

The genotypic diversity of the strains identified as *X. euvesicatoria* was evaluated by RAPD analysis. Four random primers were used for initial experiments. CUGEA-3, CUGEA-4 and CUGEA-5, and they did not give differentiation between the strains. Therefore, were excluded from further analysis.

**Figure 4.**
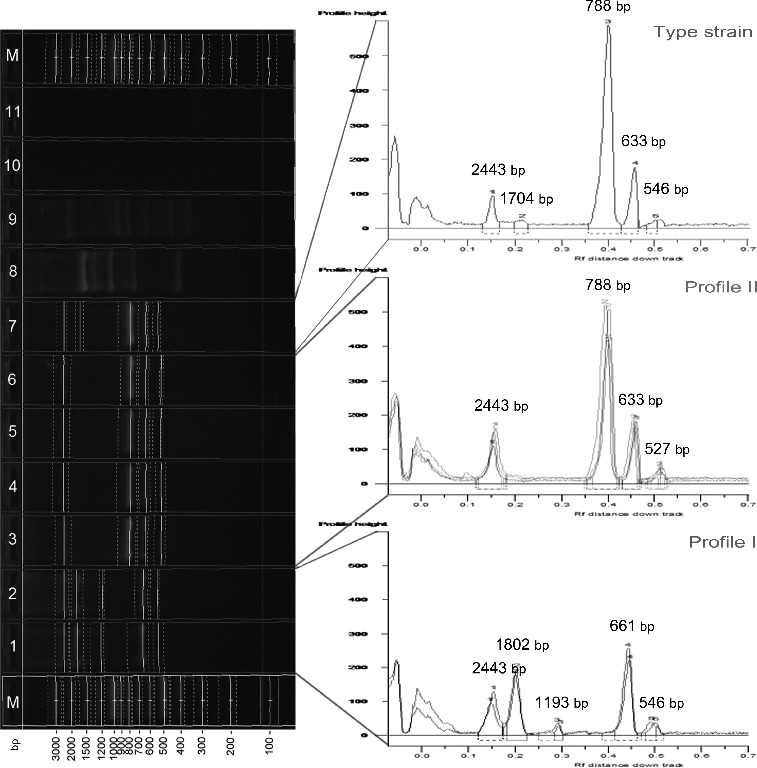
RAPD analysis with primer CUGEA-6. On the right: M – DNA ladder, 1, 2 – representative *X. euvesicatoria* strains forming profile I, 3–6 – representative *X. euvesicatoria* strains forming profile II, 8 – *X. vesicatoria*, 9 – *X. gardneri*, 10 – *X. perforans*, 11 – PCR mix. On the left: graphs of the two profiles and the profile of the type strain *X. euvesicatoria* NBIMCC 8731. The numbers on the top of the graphs correspond to the length of the amplicons.

Amplification with CUGEA-6 differentiated the studied *X. euvesicatoria* strains in two distinct groups according to their RAPD profiles. The first group included 10 strains, all of which Bulgarian (36%), and its profile consisted of five products (profile I). The second and larger group comprised 34 strains – 64% of the Bulgarian and all of the Macedonian strains and its profile possessed four products (profile II). The groups shared equal length of the largest product (2443 bp) and disclosed similar lengths of the two smallest products (661 bp/633 bp and 548 bp/ ∼527 bp). Two of the PCR products of profile I and one of the PCR products of profile II were clearly distinguishable (Figure 4). The type strain's profile was characterized by five products and it was observed to be a mixture between profiles I and II. It possessed the largest product of 2443 bp, the smallest one of profile I (548 bp) and two other equal to profile II. The fifth product's length was very close to the length of one of the products of profile I (Figure 4).

### Sub-species metabolic diversity

**Figure 5.**
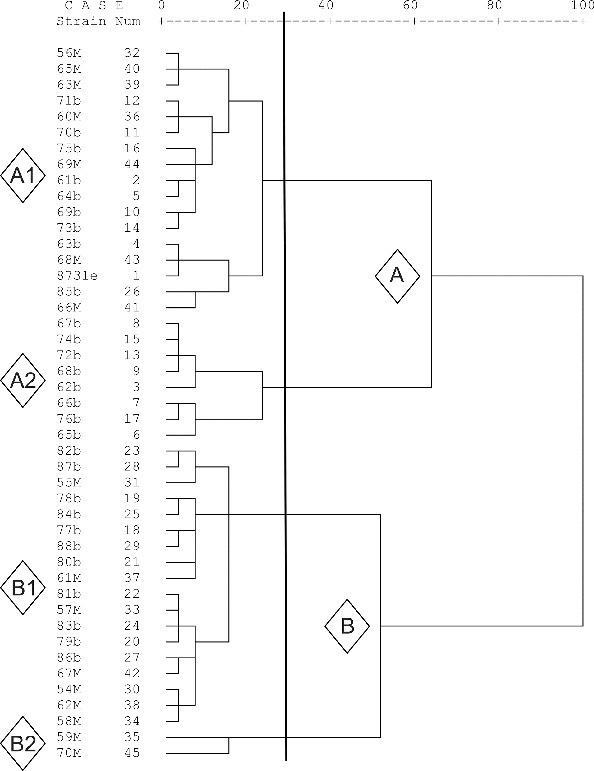
Distribution of the *X. euvesicatoria* strains according to the similarity of their Biolog metabolic profiles.

The phenotypic diversity of the strains was evaluated by Biolog^TM^ system. The metabolite profiles of the strains showed similarity in 45 of the substrates. All the strains utilized 15 carbon sources and did not utilize 30 of them. The Bulgarian strains did not utilize another 10 additional substrates. The Macedonian strains did not utilize overall 33 substrates and utilized 19 substrates. The processing of Biolog^TM^ data separated the tested strains into two main clusters A and B the larger of which included 61% of the Bulgarian and 44% of the Macedonian strains (Figure 5). At 75% similarity four sub-clusters were formed (A1, A2, B1 and B2). A1 and B1 were mixed of Bulgarian and Macedonian strains. A2 sub-cluster comprised only Bulgarian strains and B2 sub-cluster was the smallest one with only two Macedonian strains.

### Sub-species resistance diversity

**Figure 6.**
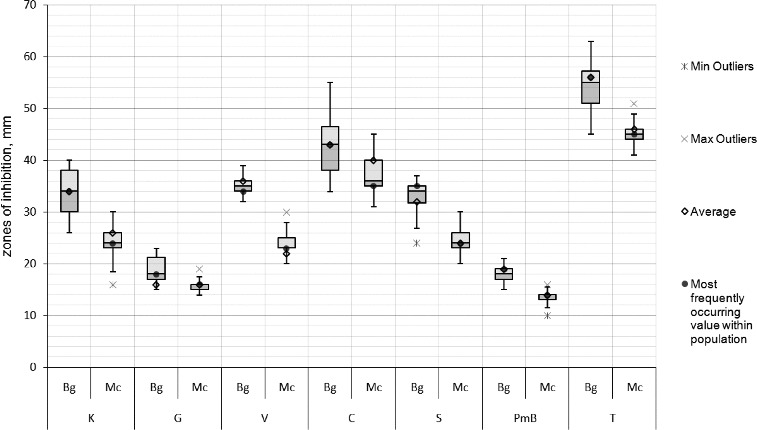
Box-plot of the zones (mm) of the susceptibility to antibiotics of the *X. euvesicatoria* strains. Bg – Bulgarian strains, Mc – Macedonian strains, K – kanamycin, G – gentamycin, V – vankomycin, C – chloramphenicol, S – streptomycin, PmB – polymixin B sulphate and T – tetracycline.

The susceptibility of the strains to 12 antibiotics was determined. All strains were resistant to cefazolin, sulfomethoxazole-trimetoprim, bacitracin, lincomycin, and ampicillin and susceptible to kanamycin, gentamycin, vancomycin, chloramphenicol, streptomycin, polymyxin B sulphate and tetracycline. The inhibition zones of the Bulgarian strains were statistically proven to be considerably larger than the Macedonian ones in the tests with kanamycin, streptomycin, polymyxin B sulphate, tetracycline and vankomycin (Figure 6). All strains showed a great susceptibility to chloramphenicol and tetracycline according to the limit values of NCCLS. The data obtained corresponded to the results of Shenge et al. [29] for the susceptibility of xanthomonads to streptomycin, gentamycin, and chloramphenicol.

**Figure 7.**
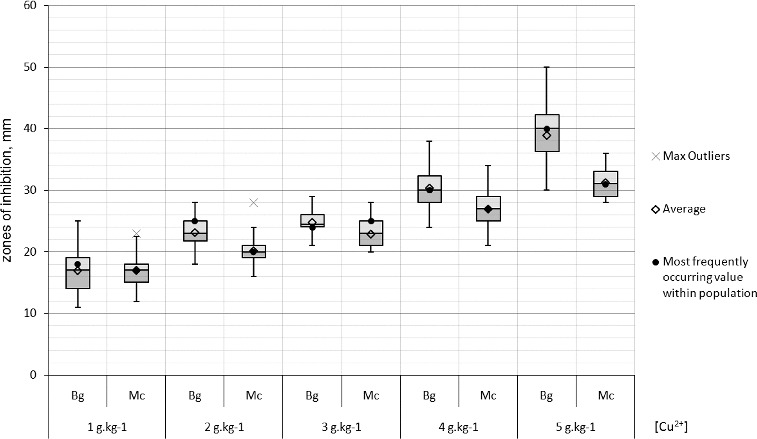
Box-plot of the zones (mm) of the susceptibility to copper of the *X. euvesicatoria* strains. Bg – Bulgarian strains and Mc – Macedonian strains.

Resistance in populations to copper ions was not observed. Two-third of the strains were weakly susceptible to 1 g kg^−1^ copper ions. All strains were strongly susceptible to copper concentrations 3–5 g kg^−1^. The inhibition zones of the Bulgarian strains were statistically proven to be relatively larger than the Macedonian ones in the copper tests (Figure 7).

The two groups of strains according to their RAPD profiles showed some relativity to the groups formed by the metabolic patterns. All the strains with RAPD profile I were located in metabolic cluster A and with the exception of two strains formed the homogenous A2 sub-cluster. The strains from profile II were relatively equally distributed among A1 and B1 metabolic sub-clusters with only two strains constituting the small B2 sub-cluster. The Bulgarian strains were distributed in the three larger Biolog metabolic sub-clusters and in the two RAPD profiles while the Macedonian strains were mainly separated in two of the larger Biolog metabolic groups and corresponded to a single RAPD profile.

**Figure 8.**
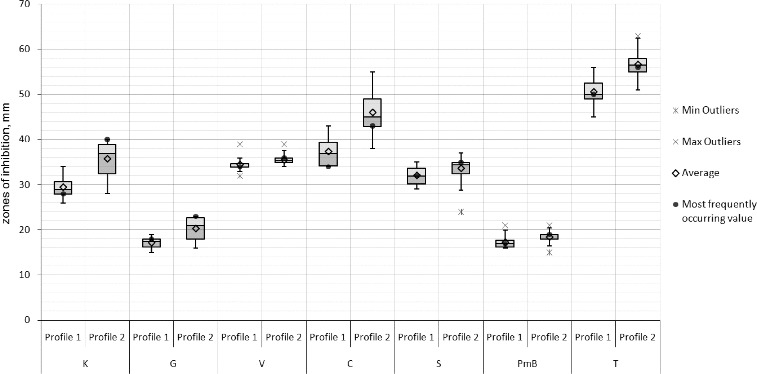
Box-plot of the zones (mm) of the susceptibility to antibiotics of the *X. euvesicatoria* strains from Bulgaria according to their RAPD profiles. K – kanamycin, G – gentamycin, V – vankomycin, C – chloramphenicol, S – streptomycin, PmB – polymixin B sulphate and T – tetracycline.

**Figure 9.**
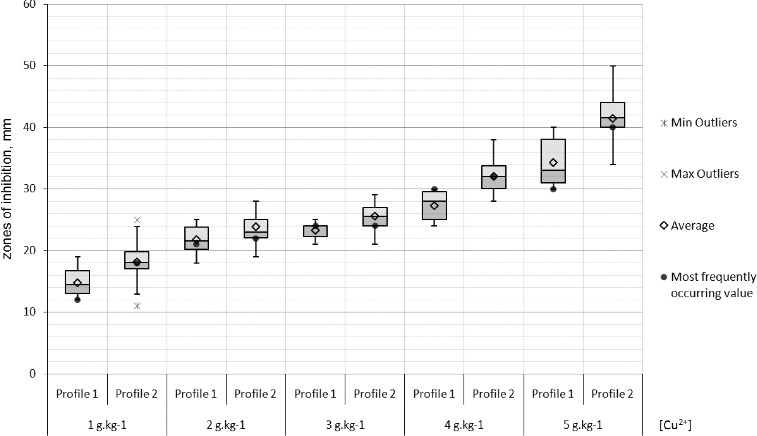
Box-plot of the zones (mm) of the susceptibility to copper of the *X. euvesicatoria* strains from Bulgaria according to their RAPD profiles.

Differences between the Bulgarian and Macedonian populations were clearly observed in the antibiotics and copper tests. The resistance variation intervals (in mm) to gentamycin, tetracycline, and 5 g kg^−1^ copper ions were comparatively narrower with the Macedonian strains. The comparative analyses of the strains forming the two RAPD profiles did not show any differences concerning the susceptibility to antibiotics and the susceptibility to copper which could be expected as RAPD profile II comprises strains from both populations. However, the statistical analysis of only the Bulgarian strains revealed that the strains from RAPD profile II were relatively more susceptible to kanamycin, gentamycin, chloramphenicol and tetracycline than the strains from RAPD profile I (Figure 8). Moreover, the larger part of the Bulgarian strains from RAPD profile II were comparatively more susceptible to copper than the rest forming RAPD profile I (Figure 9).

**Figure 10.**
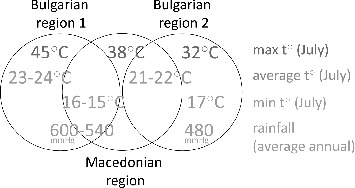
Basic climate conditions of the regions ancestor for the studied strains.

Some interesting facts concerning the origin of the strains and their grouping in the Biolog metabolic clusters can be observed. The Bulgarian strains originate from regions with two types of climatic conditions. The average temperatures in July of the first type vary between 23–24 °C with maximums up to 45 °C and minimums of ∼16 °C, and the average annual rainfall is 540–600 mmHg.[30–32] All Bulgarian strains from Biolog metabolic cluster A were isolated from pepper plants grown in these regions. The second type is characterized by average temperatures in July between 21 and 22 °C, maximums up to 32 °C, minimums of ∼17 °C, and average annual rainfall of 480 mmHg.[30–32] and it is the origination of the Bulgarian strains from Biolog metabolic cluster B. The Macedonian strains are evenly distributed between the two Biolog clusters A and B and originate from a single region in Macedonia. This region seems to share characteristics of both the Bulgarian climatic types – average temperatures in July of 21 °C, minimums of 15 °C, maximums up to 38 °C, and average annual rainfall of ∼540 mmHg.[32,33] (Figure 10). The pepper varieties did not have any relation to the observed RAPD profiles, Biolog metabolic clusters, or susceptibility to antibiotics and copper. The metabolic patterns might be a result of the adaptation of the bacteria to the environmental conditions.

## Conclusions

Based on our studies on the RAPD analysis, antibiotics and copper tests, the Macedonian population of *X. euvesicatoria* manifested a relative homogeneity while a greater diversity was observed in the Bulgarian population. The Bulgarian population was more susceptible to some antibiotics, including streptomycin and tetracycline, and relatively more susceptible to copper.
